# Shaoyao Gancao Decoction: a comprehensive review of modern clinical applications and underlying pharmacological mechanisms

**DOI:** 10.3389/fphar.2025.1656217

**Published:** 2025-10-20

**Authors:** Du Hong, Ying Xiao

**Affiliations:** The First Affiliated Hospital of Zhejiang Chinese Medical University (Zhejiang Provincial Hospital of Traditional Chinese Medicine), Hangzhou, Zhejiang, China

**Keywords:** sgd, TCM, clinical application, pharmacological mechanism, modern research

## Abstract

Shaoyao Gancao Decoction, a classic formula from Zhang Zhongjing in the Han Dynasty, has the effects of regulating the function of the liver, relieving spasm and stopping pain. Traditionally, it is used to treat pain syndromes related to “stiffness and spasm of tendons and vessels”, such as abdominal pain, muscle spasm and neuralgia. Modern studies have shown that this formula has shown significant clinical efficacy in neurological diseases (such as neuralgia and neurodegenerative diseases), digestive system diseases (such as irritable bowel syndrome and gastric spasm), gynecological diseases (such as dysmenorrhea and endometriosis), and adjuvant treatment of tumors (such as pain management after chemotherapy). Its core action mechanism involves multi-target regulation, including inhibiting central nervous excitability, regulating calcium ion channels, inhibiting the release of inflammatory factors, and directly relaxing smooth muscles. This paper summarizes the current clinical applications and pharmacological advances of the Shaoyao Gancao Decoction, while exploring its application potential in emerging fields, aiming to achieve a modern breakthrough and new clinical applications of this traditional compound prescription.

## 1 Introduction

The Shaoyao Gancao decoction (SGD) is derived from “Treatise on Febrile and Miscellaneous Diseases” written by Zhang Zhongjing in the Han Dynasty. “In case of cold damage, with floating pulse, spontaneous sweating, frequent urination, restlessness, slight aversion to cold, and spasm of the feet. Using Cinnamon Twig Decoction to attack the exterior is a wrong treatment. Then use SGD, and the feet will stretch.” “Question: The symptoms resemble those of Yangdan Decoction. When treated according to the regular method, the condition gets worse, with cold limbs, dryness in the throat, spasm of the shanks, and delirium. At midnight, when yang qi returns, the two feet should become warm. If the shanks are still slightly spastic, use SGD again, and then the shanks will stretch.” It is composed of Paeonia lactiflora and Glycyrrhiza uralensis, and mainly treats spastic pain such as “spasm of the feet” and “abdominal pain”, and has the effects of nourishing the liver, relieving spasm and relieving pain (Bi et al., 2014), and is known as “Chinese Medicine morphine” (Luo et al., 2023). Paeonia lactiflora and Glycyrrhiza uralensis are formulated in a ratio of 1:1. In Japan, it is called “Shakuyaku - Kanzo - to” (Hidaka et al., 2009), and in South Korea, it is called “Jakyak - Gamcho - Tang” (Kim et al., 2024). For more than 1,000 years, in Chinese traditional Chinese medicine clinics, due to its good therapeutic effect, SGD has been widely used for various diseases such as pain (Qian et al., 2023). Modern pharmacological and clinical studies have confirmed that SGD has clinical efficacy for various painful diseases, spastic diseases, inflammatory diseases, gynecological diseases, bronchial asthma, Parkinson’s disease, and constipation (Xu et al., 2022; Shao et al., 2019).

Radix Paeoniae Alba (white peony root) is the dried root of *Paeonia tactilora* Pall. (common peony) and *P. lactiflora* Pall. var. *trichocarpa* Bunge (hairy fruit peony), serving as the “principal botanical drug” in SGD (SGD). It can be used to treat spasmodic abdominal pain, visceral pain, cancer pain, trigeminal neuralgia, chronic gastritis, chronic hepatitis B, rheumatoid arthritis, restless legs syndrome and other diseases (Li et al., 2020; Yang et al., 2024). Its chemical metabolites mainly include monoterpenes and their glycosides, triterpenoids, flavonoids, tannins, sterols, tannins and volatile oils (Yang et al., 2024; Shen et al., 2021).

GanCao is the root and rhizome of Glycyrrhiza uralensis Fisch (liquorice), Glycyrrhiza inflate Bat (inflated fruit liquorice), or Glycyrrhiza glabra L (smooth fruit liquorice), and it is also the “ministerial drug” in SGD. Liquorice has pharmacological effects such as anti–infl ammation, antioxidant, antibacterial, anti - tumor, antiviral, and neuroprotective effects (Shang et al., 2022). It plays a very important role in the treatment of digestive system diseases, respiratory system diseases, and pain relief. So far, more than 400 metabolites have been isolated from the genus Glycyrrhiza, and a systematic metabolite database has been established. According to their chemical structures, these metabolites can be classified into flavonoids, saponins, and coumarins. Among them, flavonoids and triterpenoid saponins are more abundant in the roots or rhizomes of liquorice (Cheng et al., 2021).

Traditional Chinese medicine has the advantage of multi - target regulation, and as a natural medicine, traditional Chinese medicine has relative safety. This article will summarize and analyze the pharmacological progress and clinical research of SGD, in order to explore the broader clinical effects of SGD.

## 2 Drug information

### 2.1 Metabolite analysis

Xu Yanli et al. from Gansu University of Chinese Medicine (Yan et al., 2021) conducted a qualitative analysis of the chemical metabolites of SGD using ultra-high performance liquid chromatography-quadrupole-electrostatic field orbitrap high-resolution mass spectrometry (UHPLC-Q-Exactive Orbitrap MS) technology. A total of 129 metabolites were finally identified, including 82 flavonoids (mainly including liquiritin, isoliquiritin, liquiritigenin, isoliquiritigenin, glabridin, licorice chalcone A, licorice chalcone B, apiose liquiritin, formononetin, etc.), 20 terpenoids (paeoniflorin, albiflorin, benzoylpaeoniflorin, oxypaeoniflorin, glycyrrhizic acid, etc.), 10 phenolic metabolites, 7 coumarins, and 10 other metabolites. The study systematically identified 129 compounds of SGD, which can clarify the effective components of the drug and provide direction for the secondary development of prescriptions.

### 2.2 Paeoniflorin

The content of paeoniflorin (PF) is the highest in Paeonia lactiflora. It is an effective glucoside in Paeonia lactiflora and also a major active metabolite in Paeonia plants. It has multiple effects such as spasmolysis, analgesia, sedation, antipyretic, anti - inflammatory and antioxidant (Chen et al., 2024; Zhang and Wei, 2020), anti - tumor activity (Xiang et al., 2020), antidepressant properties (Wang et al., 2021), and treatment of neurodegenerative diseases (Peng et al., 2022). Regarding pain relief, multiple clinical studies have shown that PF exerts analgesic effects through multiple pathways. Research by Ruan (Ruan et al., 2021) et al. has shown that PF partially alleviates inflammatory pain by inhibiting the activation of the TRPV1 and succinate/SUCNR1 - HIF - 1α/NLPR3 pathways, and can significantly relieve paw swelling caused by Complete Freund’s Adjuvant (CFA), as well as mechanical and thermal pain. Research by Feng (Luo et al., 2024) et al. found that PF alleviates neuropathic pain by regulating microglia - astrocyte crosstalk through the HSP90AA1/HMGB1 pathway and inhibits the inflammatory response of chronic constriction injury, which further demonstrates the potential of PF in the treatment of neuropathic pain. Fan (Fan et al., 2018) et al. studied mice that underwent plantar incision surgery and showed that paeoniflorin could inhibit the TLR4/MMP - 9/2/IL - 1β signaling pathway in microglia induced by plantar incision and suppress postoperative pain. Tsugunobu Andoh (Andoh and Goto, 2023) et al. demonstrated that PF could inhibit postoperative pain through fibroblast proliferation and accelerate wound healing at the incision site. In terms of anti - inflammation and immunomodulation, Zhang and Wei (2020) found that PF had a wide range of anti - inflammatory and immunomodulatory effects. It could regulate the function and activation of immune cells, reduce the production of inflammatory mediators, and restore abnormal signaling pathways. Gao et al. (2015) investigated the effects of paeoniflorin on cytochrome P450 (CYP) 3A4 and CYP2D6 in human hepatocellular carcinoma HepG2 cells, and found that paeoniflorin has a regulatory effect on the mRNA expression of CYP3A4 and CYP2D6.PF also has a mitigating effect on cardiac hypertrophy by regulating oxidative stress and the Nrf2 signaling pathway *in vitro* and plays a wide range of roles in cardiovascular diseases (Ren et al., 2023).

### 2.3 Licorice

Licorice is an metabolite of SGD, a traditional Chinese medicine preparation. The main active metabolites of licorice can be divided into three categories: three types of terpenoids (glycyrrhizic acid, glycyrrhetinic acid, and liquiritigenin), flavonoids (isoliquiritin, licoflavanone, etc.), and polysaccharides. Triterpenoids and flavonoids have a variety of pharmacological effects, similar to adrenal cortical hormones, and have anti - ulcer, antispasmodic, anti - inflammatory, anti - allergic, antiviral, detoxifying, antitussive, expectorant, and anti - tumor effects (Ji et al., 2024; Liu et al., 2023).

#### 2.3.1 Terpenoids

Terpenoids include glycyrrhizic acid, glycyrrhetinic acid and liquiritigenin. Glycyrrhizic acid and glycyrrhetinic acid have relatively strong physiological activities and possess characteristics (Xiao, 2022). Glycyrrhizic acid has unique therapeutic advantages in many aspects such as anti-tumor (Xiao et al., 2024), anti-virus (Sun et al., 2019), and promoting wound repair (Qian et al., 2022). Research by Cheng (Cheng et al., 2024) et al. suggests that glycyrrhizic acid regulates the nuclear translocation of YAP by inhibiting the Hippo/YAP signaling pathway, thereby reducing myocardial ischemia/reperfusion (MI/R) injury. This discovery may provide a novel treatment strategy for the treatment of MI/R. Research by Sun (Sun et al., 2018) et al. shows that liquiritigenin inhibits microglial activation-mediated inflammatory response by blocking the HMGB1-TLR4-NF-κB pathway, thus improving inflammatory pain. Glycyrrhizic acid coumarin extracted from licorice can be used as an effective antispasmodic drug in clinical applications by inhibiting phosphodiester-3 (Sato et al., 2006).

#### 2.3.2 Isoliquiritigenin

The experiment isolated the potent relaxant metabolite, isoliquiritigenin, from the aqueous extract of licorice. This metabolite exhibited inhibitory effects on contractions induced by various stimulants such as CCh, KCl, and BaCl2. However, the content of isoliquiritigenin in the aqueous extract of licorice was extremely low. When the aqueous extract of licorice was treated with naringinase, the glycoside metabolites (such as isoliquiritin) that were abundant but had weak activity were significantly reduced, while the content of isoliquiritigenin increased. At this time, the antispasmodic activity of the treated sample was significantly enhanced. These results indicate that isoliquiritigenin can be transformed from its glycoside metabolites and exert a potent relaxant effect in the lower part of the intestine (Sato et al., 2007).

### 2.4 Synergistic effect

PF and GA are two typical active metabolites in SGD that relieve pain (Xu et al., 2013). After transdermal administration of GA and PF to mice with simulated dysmenorrhea, Xue et al. (Ding et al., 2016) found that GA - PF could relieve pain to the same extent as meloxicam. Guan et al. (Guan et al., 2014) found through research that SGD and PF could significantly reduce the production of prostaglandin E_2_ (PGE_2_) and prostaglandin F_2_α (PGF_2_α), and significantly inhibit the mRNA expression levels of estrogen receptor α (ER - α) and oxytocin receptor (OTR), revealing the potential mechanism of action of SGD in the treatment of adenomyosis and providing a scientific basis for the ethno - pharmacological application of this traditional formula. M Kimura (Kimura et al., 1984) used frog isolated sciatic nerve - sartorius muscle specimens and mouse isolated/in - situ phrenic nerve - diaphragm specimens to explore the combined effects of PF from paeony, the main metabolite of SGD, and GLR from licorice, the main metabolite of licorice. It was found that when paeoniflorin and glycyrrhizic acid were used in a 1:2 weight ratio (consistent with the ratio in SGD), the effect was optimal.

### 2.5 Pharmacokinetics

The results of human pharmacokinetic trials are also of great significance for understanding the action mechanism of SGD, verifying the active metabolites predicted by basic research, and carrying out future pharmacokinetic and safety studies. Chiharu Sadakane et al. (2015) administered oral SGD at 2.5 or 5.0 g per day to healthy Japanese volunteers, and detected relevant target metabolites in the subjects’ plasma. All target metabolites were detected in plasma after oral administration of SGD, among which paeoniflorin, albiflorin, licorice coumarin, and isoliquiritigenin were detected in the early stage. The maximum plasma concentrations of licorice coumarin, isoliquiritigenin, and glycyrrhetinic acid, as well as the area under the plasma concentration-time curve of glycyrrhetinic acid, showed a linear relationship. This is the first demonstration in humans that these metabolites are absorbed into the bloodstream after oral administration of SGD. By comparing the pharmacokinetics of 10 bioactive metabolites in two different combinations of SGD (Radix Paeoniae Alba and Radix Glycyrrhizae), XU et al. believed that increasing the proportion of Radix Paeoniae Alba could inhibit the conversion of glycyrrhetinic acid by competing with the metabolism of glycyrrhizic acid by intestinal flora (or β-glucosidase), thereby significantly improving the bioavailability of glycyrrhizic acid, albiflorin, oxypaeoniflorin, isoliquiritin and formononetin and prolonging the duration of drug effect, and ultimately enhancing the antispasmodic and analgesic effects (Xu et al., 2013). SGD enhances the expression of CYP3A4 (liver metabolic enzyme) and MDR1 (multidrug resistance protein 1) through the pregnane X receptor pathway (Feng et al., 2018).

The co - administration of histamine H2 - receptor antagonists (cimetidine) and anticholinergic drugs (scopolamine butylbromide) with SGD has no effect on the area under the plasma concentration - time curve (AUC) of glycyrrhizic acid (GA), an active metabolite derived from glycyrrhizin in SGD. However, in the treatment of peptic ulcers, the combined use of antibacterial synthetic drugs such as amoxicillin and metronidazole (AMPC - MET) significantly reduces the bioavailability of PF in SGD and significantly decreases the AUC of GA. Therefore, the triple - therapy using antibiotics and SGD simultaneously for the treatment of chronic ulcers may not be an appropriate method (He et al., 2001). Through further research, HE et al. found that during the combined treatment of amoxicillin and metronidazole (AMPC - MET), starting to repeatedly administer SGD 1 or 2 days after the treatment to accelerate the recovery of the decreased bioavailability of PF in SGD may be clinically useful. Similar dosing regimens may also be useful in other combination therapies involving traditional Chinese medicine formulas and antibacterial synthetic drugs to ensure the efficacy of bioactive glycosides in the formula (He et al., 2003). It can be speculated that Western medicines may affect the blood drug concentration of traditional Chinese medicine preparations, but repeated dosing can improve bioactivity.

## 3 Clinical research evidence

### 3.1 Analgesia

Pain can be classified into nociceptive pain (acute trauma, postoperative pain), inflammatory pain, neuropathic pain, and functional pain (fibromyalgia). SGD is used for controlling postoperative wound pain by reducing the secretion of prostaglandins, a pain-causing factor (Nishijima and Nishimura, 2015). For patients with endometriosis and adenomyosis who wish to conceive, the SK/TS cycle therapy can serve as a conservative anti-dysmenorrhea therapy (Tanaka, 2003). In a study of 30 perimenopausal women with uterine fibroids, after conservative treatment with SGD, more than 60% of patients with fibroids smaller than a fist size (with dysmenorrhea and/or main complaint of dysmenorrhea) showed some degree of improvement in dysmenorrhea after traditional Chinese medicine treatment, while those with fibroids larger than a fist size showed no significant improvement (Sakamoto et al., 1998). The production of prostaglandins (PG) in the myometrium increases during menstruation. SGD, licorice, and GA (glycyrrhetinic acid) inhibit the release of [14C]-AA in a dose-dependent manner. This is the first discovery that SGD exerts an analgesic effect by inhibiting cPLA2 activity to suppress PG production in the myometrium (Shibata et al., 1996). SGD can also counteract dysmenorrhea by inhibiting arachidonate conversion in the endometrium to reduce prostaglandin levels (Imai et al., 1995).

Using streptozotocin-induced diabetic mice as the research object, SGD significantly increased the nociceptive threshold of diabetic mice. Moreover, the antinociceptive effect of SGD in diabetic mice is not mediated by the opioid system. This effect is achieved by selectively activating the spinal descending inhibitory α2 - adrenergic system without activating the serotonergic system. The enhanced analgesic mechanism mediated by the α2 - adrenergic receptor in the spinal cord of diabetic mice suggests that SGD exerts an analgesic effect by activating descending noradrenergic neurons (Omiya et al., 2005). SGD exerts an analgesic effect through a down - regulating effect on the TRPV1 channel in the rat model of arthritic pain (Sui and Tang, 2016). The analgesic effect of SGD may be related to the inhibition of the over - expression of Sirt1 (Zhang et al., 2013).

### 3.2 Digestive system

Professor Yuan Yiqing and his team found that SGD (SGD) with modifications for treating acute gastric ulcer can effectively alleviate clinical symptoms, reduce inflammatory response, increase the expression levels of epidermal growth factor (EGF) and epidermal growth factor receptor (EGFR) in gastric mucosa, exert protective effects on gastric mucosa, thereby improving therapeutic efficacy. The drug shows good safety and holds important clinical application value (Yiqing and Xiaojun, 2021). Additionally, studies have revealed that SGD inhibits the activity of H^+^-K^+^-ATPase, thereby suppressing gastric acid secretion and improving peptic ulcer (Satoh et al., 2001). SGD also exhibits protective effects against ethanol-induced gastric ulcer in rats. It was found that SGD treatment significantly increased the levels of EGF, prostaglandin E2 (PGE2), superoxide dismutase (SOD), and B-cell lymphoma-2 (Bcl-2) in the gastric tissue of ethanol-induced gastric ulcer rats, while decreasing the levels of tumor necrosis factor-α (TNF-α), thiobarbituric acid reactive substances (TBARS), and caspase-3. These results indicate that SGD can alleviate gastric tissue cell apoptosis in ethanol-induced gastric ulcer rats (Jin et al., 2022).

Patients with diarrhea-predominant irritable bowel syndrome (IBS-D) exhibit excessive peristalsis, and antispasmodics may be useful therapeutic agents. Studies have found that SGD can inhibit colonic peristalsis, among which glycyrrhizin and isoglycyrrhetinic acid are particularly relevant in its metabolites (Kobayashi et al., 2024). Spraying the SGD solution directly into the duodenum can inhibit duodenal peristalsis (Fujinami et al., 2017). Direct application of SGD to the duodenal papilla can significantly inhibit the increase in serum amylase levels (Fujinami et al., 2015). During endoscopic retrograde cholangiopancreatography, the antispasmodic effect of directly spraying SGD on the duodenal wall was confirmed (Sakai et al., 2009). Similarly, direct spraying of SGD on the colonic mucosa can inhibit colonic spasm. Therefore, SGD may be useful during colonoscopy when the use of anticholinergic drugs is contraindicated (Ai et al., 2006).

SGD alleviates *Helicobacter* pylori-induced chronic atrophic gastritis (CAG) by inhibiting MAOB (Li Z. et al., 2024), and *Helicobacter pylori* plays an important role in CAG. SGD treatment can reduce CAG-induced gastric mucosal damage, decrease apoptosis of gastric mucosal epithelial cells, and inhibit the inflammatory response. SGD affects the function of the Oddi sphincter (SO) in hypercholesterolemic rabbits by protecting the interstitial cells of Cajal - smooth muscle cell network in the enteric nervous system. Compared with the model group, the morphology and ultrastructure of SO in the SGD group were repaired. In addition, the levels of protein gene product 9.5 (PGP9.5), NO, SMCs, and ICC were significantly increased, while the level of substance P (SP) was significantly decreased. Meanwhile, SGD may treat SO dysfunction by upregulating the expression of c-Kit and SCF and activating the stem cell factor (SCF)/c-Kit signaling pathway. This pathway restores SO by upregulating the expression of Bcl2 and inhibiting the expression of cleaved caspase-3, Bax, and tumor necrosis factor (Zhu et al., 2021).

SGD is a well - known traditional Chinese medicine formula for the treatment of liver injury. Chemical liver injury is closely related to gut microbiota and their metabolites. This study investigated the changes of gut microbiota, fecal metabolites, and short - chain fatty acids (SCFAs) in CCl4 - induced liver injury in SD rats, as well as the therapeutic effects of SGD. The results showed that CCl4 - induced liver injury led to overexpression of CYP2E1, enhanced oxidative stress, decreased antioxidant enzymes (SOD, GSH), increased peroxidation product MDA and inflammatory response (IL - 6, TNF - α), and SGD treatment could improve these conditions. Hematoxylin - eosin (HE) staining indicated that SGD could alleviate liver tissue lesions, which was also confirmed by the recovery of liver index, ALT, and AST. Gut microbiota plays a protective role in the pathogenesis of liver injury and has positive significance for the efficacy of SGD. In addition, SGD can treat liver injury by regulating gut microbiota, their metabolites, and SCFAs. This provides useful evidence for studying the pathogenesis of liver injury and the clinical application of SGD (Li et al., 2022). The therapeutic effect of SGD on liver injury and its metabolic mechanism were studied using 1H NMR and UPLC - MS. Changes in the levels of biomarkers revealed the therapeutic effect of SGD on liver injury and were of great significance for inferring the possible metabolic mechanism (Sun et al., 2020). SGD has been proven to have a good hepatoprotective effect. Pharmacokinetic studies have shown that paeoniflorin, paeoniflorin, oxypaeoniflorin, liquiritin, isoliquiritin, liquiritin, formononetin, ononin, glycyrrhizic acid, and glycyrrhetinic acid are potential active metabolites of SGD in the treatment of acute liver injury (Li X. et al., 2024).

### 3.3 Gynecology

It is known that amenorrhea, oligomenorrhea, irregular menstrual cycles, luteal insufficiency, and infertility are often associated with hyperandrogenemia. SGD can reduce the high serum testosterone levels in women with oligomenorrhea or amenorrhea, and some of these infertile women are able to conceive (Takeuchi, 1988). One of the mechanisms by which SGD reduces serum testosterone levels is by directly acting on the ovaries, stimulating aromatase activity, leading to a decrease in serum testosterone secretion, which is the additive effect of Paeonia lactiflora and Glycyrrhiza uralensis (Takeuchi et al., 1989). In a study of ovariectomized (OVX) rats, it was found that SGD can increase the content of the estrogen precursor DHEA-S in the serum of ovariectomized rats (Kato and Okamoto, 1992). At the same time, paeoniflorin, glycyrrhizic acid, and glycyrrhetinic acid, by directly acting on the pre-estrus ovaries of rats, affect the conversion of delta 4-androstenedione to testosterone, inhibit testosterone synthesis, stimulate aromatase activity, and promote estradiol synthesis (Ta et al., 1991). SGD can treat risperidone-induced amenorrhea (Yamada et al., 1999).

SGD has the effect of treating polycystic ovary disease (Takahashi and Kitao, 1994). Explore the mechanism of action of SGD in the treatment of polycystic ovary syndrome (PCOS). SGD can effectively regulate the estrous cycle of PCOS rats, reduce body weight and blood lipid levels. SGD significantly remodels the gut microbiota structure, especially the ba - related microbiota, and can significantly change the mRNA expression of genes related to the BAs metabolic pathway. The above results were verified by fecal microbiota transplantation in SGD rats. Therefore, SGD may improve dyslipidemia in PCOS rats by remodeling the gut microbiota structure and regulating the bile acid/FXR pathway (Duan et al., 2025). In 20 infertile Japanese patients with polycystic ovary syndrome, we used SGD to reduce plasma testosterone levels, thereby inducing pregnancy (Takahashi et al., 1988).

Peony and licorice can be used as antispasmodics to inhibit the contraction of uterine smooth muscle in pregnant rats (Sumi et al., 2015). It has at least two steps of inhibitory effects on myometrial contraction. This inhibitory effect is derived from licorice itself and the metabolites of glycyrrhizic acid in licorice, and it will not temporarily enhance the contraction induced by PGF2α (Sumi et al., 2014).

Human adenomyosis cells were cultured *in vitro* to study the biological activity of SGD and its potential molecular mechanisms. It was found that SGD, paeoniflorin, and liquiritin inhibited the proliferation of human adenomyosis cells and induced their apoptosis in a dose-dependent manner. SGD and paeoniflorin significantly reduced the production of PGE2 and PGF2α. In addition, they significantly decreased the mRNA levels of ER-α and OTR (Guan et al., 2014). In the experimental animal model of SHN mice, we examined the effect of SGD on the spontaneous development of uterine adenomyosis and hyperplastic alveolar nodules (HAN) in the breast. It was found that the incidence of adenomyosis in the SGD group was significantly reduced. Long-term exposure to these botanical drugs had no significant effect on serum prolactin (PRL) levels, estrous cycle, food intake, and body growth. Therefore, the current mouse data suggest that oral administration of these botanical drugs is a useful tool for the treatment of uterine adenomyosis or breast diseases such as cystic mastitis (Mori et al., 1993). In Japanese women with endometriosis, adenomyosis, or leiomyoma, SGD can be recommended for the treatment of menopausal symptoms caused by gonadotropin-releasing hormone agonists without having a negative impact on serum estradiol levels (Tanaka, 2001).

We have previously demonstrated the presence of high concentrations of endothelin-1 (ET) and binding sites for atrial natriuretic peptide (ANP) in the corpus luteum and the renin-angiotensin system (RAS). The aim of this study was to determine the presence of binding sites for ET, renin, angiotensin II, and ANP in the proestrus ovary and to examine the effects of botanical medicines [Tokishakuyakusan (TS), Keishibukuryogan (KB), Shakuyakukanzoto (SK), and Unkeito (UT)] on them *in vivo*. The binding sites for ET, metabolites of the RAS, and ANP were highly expressed in ovarian tissues. TS, KB, SK, and UT decreased the ovarian ET levels, while the binding sites for RAS metabolites and ANP tended to increase. However, there were no significant changes in plasma levels of ET, renin, angiotensin II, and ANP before and after treatment with TS, KB, SK, and UT. Combining the previously observed binding sites for ET, RAS, and ANP in the ovary, we propose here a functional regulatory role of the ERAANPS (endothelin-renin-angiotensin system) in the ovary. In addition, these results suggest that TS, KB, SK, or UT may regulate the ovarian ERAANPS (Usuki et al., 1992a).

It can be seen that for common gynecological diseases, SGD effectively treats hyperandrogenism-related diseases, PCOS, abnormal uterine contractions, and benign gynecological tumors through a multi-target mechanism (hormone regulation, gut microbiota, prostaglandin pathway, etc.), and has both safety and clinical potential.

### 3.4 Nervous system

Use network pharmacology to deeply study the mechanism of action of SGD in the treatment of Alzheimer’s disease (AA). Preliminary animal experiments verify that it is closely related to the PI3K/AKT signaling pathway. This finding may provide new evidence for the clinical application of SGD in neurodegenerative diseases (Lv et al., 2022).

Selective sciatic nerve injury (SNI) neuropathic pain model rats were orally administered with SGD intervention. SGD has a significant regulatory effect on neuropathic pain, can increase the pain threshold, and reduce the levels of SP, β - EP, PGE2 and NO. Using metabolomics combined with PLSR and multi - index comprehensive methods, five metabolites, namely paeonol, dl - arabinose, benzoic acid, xilingolide A and paeonolide C, were found to be the effective metabolites of SGD in the treatment of neuropathic pain (Feng et al., 2020). SGD can also improve chemotherapy - induced neuropathic pain (Schröder et al., 2013).

The suture method was used to establish a rat model of spastic paralysis in the convalescent stage of stroke, and to explore the effects of SGD on the amino acid content and receptor expression in the brain of rats with spastic paralysis. The results showed that SGD at a ratio of 3:1 could improve the spastic paralysis state after stroke, significantly improve neurological symptoms, reduce muscle tension, and increase the pain threshold. It is suggested that SGD at a ratio of 3:1 can effectively relieve spasm and pain. The mechanism may be related to increasing the content of inhibitory amino acids and their receptor expression in rats with spastic paralysis after stroke, thereby enhancing the signal transduction of inhibitory amino acids. At the same time, although excitatory amino acids have no obvious effect, there is a decreasing trend, which can inhibit the expression of excitatory amino acid receptors, thereby weakening excitatory signal transduction and ultimately alleviating neurotoxicity. It is suggested that SGD can relieve the spastic state by regulating the balance of the neurotransmitter system, thus achieving the effect of antispasm (Wang et al., 2016).

Tetanus is an infectious disease caused by *Clostridium tetani*, which produces tetanus spasm protein. Severe tetanus requires intensive care with sedatives and muscle relaxants. However, long - term use of these drugs is associated with the occurrence of post - intensive care syndrome (PICS). Some studies have shown that SGD is effective in treating muscle spasms caused by tetanus (Nakae et al., 2017). Clinical research has found that SGD has certain clinical application value in the treatment of tetanus complicated by muscle spasms. It can reduce the use of sedatives and analgesics, and can also prevent tetanus patients who need intensive care from entering the PICU (Oshima et al., 2023).

### 3.5 Immune system

Systemic lupus erythematosus (SLE) is a chronic and devastating autoimmune disease accompanied by severe organ damage. To clarify the role of SGD in SLE, we treated female MRL/lpr mice with SGD, whose main metabolites were paeoniflorin (56.949 μg·mL-1) and glycyrrhizin (459.393 μg·mL-1). We found that SGT treatment alleviated lymphadenopathy and splenomegaly in MRL/lpr mice, reduced urine protein and anti-ds-DNA antibody concentrations, and mitigated kidney pathology. SGT could also effectively regulate the oxidation/antioxidation balance, significantly decrease the contents of malondialdehyde (MDA) and nitric oxide (NO) in MRL/lpr mice, and remarkably increase the activities of superoxide dismutase (SOD) and glutathione peroxidase (GSH-Px). After treatment with SGD, the content of neutrophil extracellular traps (NETs) in MRL/lpr mice was also reduced to a certain extent. SGT may play a good therapeutic role in SLE by improving tissue inflammatory damage caused by oxygen free radicals, thus regulating the TLR9-mediated NETosis process (Qian et al., 2023).

To explore the regulatory mechanism of the cAMP-PKA signaling pathway mediated by the traditional Chinese medicine metabolite SGD on the levels of aquaporin 5 (AQP5) and muscarinic receptor 3 (M3R) in Sjogren’s syndrome (SS). Compared with normal mice, the body weight, 5-min saliva secretion, 30-min tear secretion, and tear film break-up time of model mice decreased from 1 to 6 weeks after immunization (all P < 0.05), and the water intake increased (all P < 0.05). In the model group of rats, the submandibular gland glands atrophied, accompanied by glandular acini of different sizes, reduced numbers, loose arrangement, ductal dilation, and varying degrees of lymphocyte infiltration. The conditions of mice in the SGD group were all improved. The positive expressions of AQP5 and M3R in acinar cells of each SGD dose group were higher than those in the normal group (Wang et al., 2020). It can be seen that SGD has positive significance for the treatment of Sjogren’s syndrome.

### 3.6 Musculoskeletal system

SGD can effectively alleviate the clinical symptoms of patients with osteoarthritis (OA) and improve the level of inflammation (Mitsumoto et al., 2023). SGD alleviates OA cartilage degeneration and reduces ECM degradation by upregulating COL2A1 and downregulating MMP-13. 120 key targets were screened out from the differentially expressed genes through RNA-Seq. Based on further bioinformatics analysis, interleukin 17 receptor B (IL-17RB), interleukin 23 receptor, and growth differentiation factor 5 were finally screened out as the core targets. IL-17RB has been rarely reported in previous OA studies and is worthy of further research. Subsequently, we found that the gene and protein expression of IL-17RB in the model group was significantly reversed after SGD treatment. In addition, SGD can inhibit the release of inflammatory factors in OA by mediating IL-17RB (Hou et al., 2025). Formononetin, one of the active metabolites of SGD, may exert a protective effect against OA by inhibiting the PI3K/AKT and NF-κB pathways. In addition, this study suggests that calycosin is a potential candidate drug for the treatment of OA (Shi et al., 2022). The mechanism of action of SGD in alleviating chronic inflammatory pain by regulating Sema3G protein in the dorsal root ganglia (Lin et al., 2024). The self - formulated Ye’an Zhentong Decoction/Jiawei SGD has a significant curative effect in the treatment of restless legs syndrome (RLS), can significantly improve the clinical symptoms of patients, reduce the severity of RLS, and improve the quality of life and sleep quality (Zhou et al., 2024). Our research results show that SGD can inhibit tonic contraction *in vivo*, and due to the effect of its active metabolites, G. radix is the main antispasmodic metabolite, thus supporting the traditional use of SGD. It is further proposed that SGD containing antispasmodic root botanical drug and antinociceptive root botanical drug is a good drug choice for the treatment of muscle spasms, because the treatment requires a two - pronged approach, that is, inhibiting the over - excited skeletal tissue and regulating the pain associated with spasms (Lee et al., 2013). A clinical trial study on 30 healthy adult men found that SGD has a clinical effect in the treatment of delayed - onset muscle soreness (Han et al., 2020). SGD may be effective for muscle spasms in patients with liver cirrhosis or lumbar spinal stenosis (Ota et al., 2020). It inhibits IKur in a concentration - dependent manner, and may restore the K (+) balance inside and outside the cells by inhibiting IKur and reducing K (+) efflux, while the Na (+)-K (+) pump promotes the influx of K (+) into muscle fibers. Therefore, the extracellular space of muscle fibers can reduce the excessive K (+), which may be part of the mechanism by which SGD improves muscle pain (Suganami et al., 2014). SKT may become a first - line drug for the conservative treatment of thoracic outlet syndrome (TOS) (Kubota and Miyata, 2005). At the same time, further research has found that while SGD improves muscle spasm pain, it does not have typical severe side effects such as muscle weakness and central nervous system (CNS) depression (Kaifuchi et al., 2015).

### 3.7 Skin

SGD shows good therapeutic effects on imiquimod-induced psoriasis mice. The research results suggest that the main form of SGD’s action is the SAN formed by the aggregation of active metabolites during the decoction process. It can treat psoriasis in mice by reducing the expression levels of multiple inflammatory factors, promoting normal differentiation of keratinocytes, and decreasing inflammatory cell infiltration (Qin et al., 2023).

### 3.8 Kidneys

SGD can promote the spontaneous excretion of stones, with 91% of small stones (5 × 5 mm and below) and 33% of medium-sized stones (6 × 10 mm and below) being discharged (Washizuka et al., 1983). SGD (SGD) significantly inhibits the substrate uptake activity of URAT1, OAT1, and OAT3 without exhibiting cytotoxic effects. The inhibition of substrate uptake activity of renal transport proteins suggests their mechanism of action as renal protectants (Lee et al., 2018). Additionally, SGD can improve muscle spasm relief in hemodialysis patients (Hyodo et al., 2006). Oral administration of SGD has a positive effect on muscle spasms in maintenance hemodialysis patients (Hinoshita et al., 2003).

### 3.9 Cardiovascular system

A Japanese study shows that a statistical analysis was conducted on 2,547,559 patients hospitalized in 1,798 hospitals due to acute cardiovascular diseases (acute myocardial infarction, heart failure, pulmonary embolism, or aortic dissection) from 2010 to 2021. It was found that the use of Kampo medicines increased threefold from 2010 (4.3%) to 2021 (12.4%), and Shaoyao Gancao ranked third (Isogai et al., 2024).

SGD has a cardioprotective effect. Using neonatal rat ventricular myocytes, we evaluated the direct effect of SGD on myocardial hypertrophy. SGD significantly alleviated angiotensin II (Ang II)-induced cardiomyocyte hypertrophy and cell death, and also reduced the elevated [Ca2+]i and ROS production associated with this condition. In addition, the combined application of the L-type calcium channel (L-Ca2+) blocker nifedipine indicated that SGD could antagonize the effect of L-Ca2+. These results suggest that SGD plays a protective role against Ang II-induced cardiomyocyte hypertrophy by inhibiting the L-Ca2+-mediated pathway. Therefore, this study highlights the potential of SGD for cardiac applications and paves the way for new HF prevention and treatment strategies (Tagashira et al., 2024). We have previously proposed the ovarian endothelin-angiotensin-atrial natriuretic peptide system (ERAANPS), and SGD may regulate the ERAANPS system (Usuki et al., 1992b).

### 3.10 Endocrine diseases

A clinical report described a patient with an insulinogenic index higher than the normal value. After taking a botanical medicine named SGD, this patient experienced ovulation and pregnancy. We believe that it may be useful to identify the insulin-resistant subgroup in women with PCOS, as this resistance may be important in subsequent and future explorations (Takahashi et al., 2007). The effectiveness of (shakuyaku-kanzo-to) on neuroleptic-induced hyperprolactinemia (Yamada et al., 1997).

### 3.11 Mental illness

**FIGURE 1:**
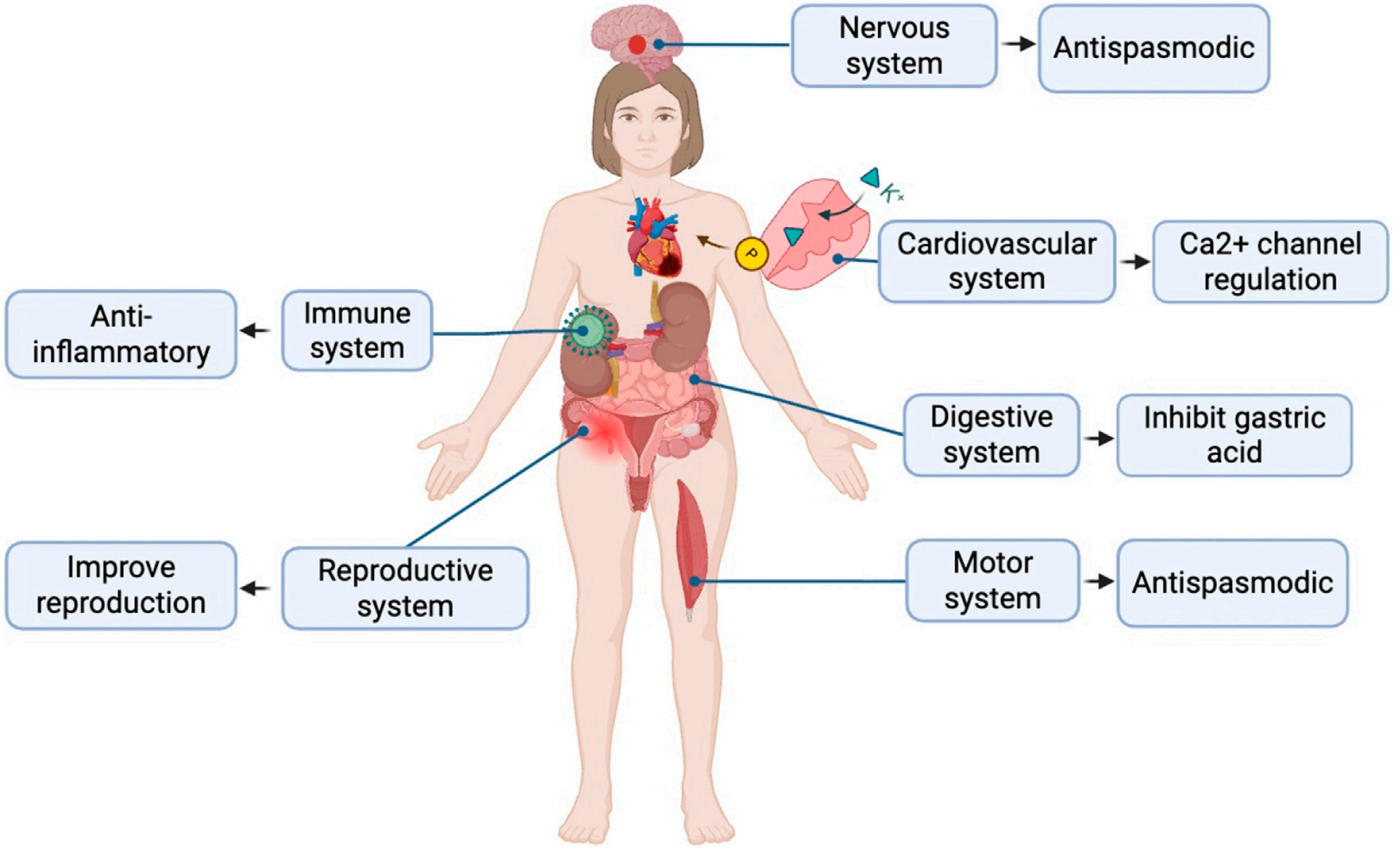
The role of shaoyao gancao decoction in various systems.

Calculate and analyze the pathogenic genes of depression through bioinformatics methods, and deduce and predict the key genes of SGD in treating depression through the correlation study with the targets of SGD. By making LPS-induced depression model mice, drug treatment, behavioral tests and detection of hippocampal tissue samples, it was found that SGD can regulate the levels of IL-10, TNF-α, BDNF, SMAD3, FGFR1 and FGFR2 to improve the depressive state, which can provide a theoretical basis for exploring the efficacy of SGD in treating depression (Li L. et al., 2024). The role in the individual systems is shown in Figure 1.

## 4 Multi - target pharmacological effects

### 4.1 Neuroprotective effects

#### 4.1.1 Regulation of neuromuscular signal transmission

This will be introduced from the aspects of action potential inhibition and synergistic mechanisms. An action potential is a rapid and reversible electrical signal transmission process that occurs on the membrane of neurons or muscle cells, and its core relies on voltage-gated ion channels. d-Tubocurarine is the first non-depolarizing muscle relaxant applied clinically, and its core mechanism is the competitive antagonism of the N2-type acetylcholine receptor. In animal experiments, it was found that SGD can inhibit the action potential of frogs (Matsushita et al., 2016). In the study of the combined effects of paeoniflorin (PF), the main metabolite of Radix Paeoniae Alba, and glycyrrhizin (GLR), the main metabolite of Radix Glycyrrhizae, on the isolated sciatic nerve-gastrocnemius muscle preparation of frogs or the isolated *in-situ* phrenic nerve-diaphragm muscle preparation of mice, it was found that the combined use of PF and GLR can block the twitch caused by indirect stimulation. At concentrations where no blocking effect was produced when used alone, the optimal synergistic ratio was 1:2 (PF:GLR). In addition, GLR also has a synergistic effect on paeonolide, oxypaeoniflorin, and succinylcholine contained in the roots of Radix Paeoniae Alba. GLR does not increase the blocking effect of d-tubocurarine. In summary, PF and GLR have a pharmacological mixed effect (Kimura et al., 1984).

#### 4.1.2 Anti-neuroinflammation and improvement of alzheimer’s disease

NLRP1 and NLRP3 are core sensor proteins of the inflammasome, belonging to the NOD-like receptors (NLRs) family, and play a key role in innate immune defense, inflammatory diseases, and autoimmunity (Barnett et al., 2023). SGD exerts neuroprotective and cognitive improvement effects by reducing NLRP1 and NLRP3 in Alzheimer’s disease cell and mouse models (Chiu et al., 2021). Beta-amyloid protein plays a major role in the neurodegeneration of Alzheimer’s disease. The accumulation of misfolded Aβ causes oxidative stress and inflammatory damage, leading to apoptotic cell death. In Aβ-gfp SH-SY5Y cells (human neuroblastoma cells), SGD reduced Aβ aggregation and the production of reactive oxygen species (ROS), and promoted neurite outgrowth. When cells expressing Aβ-gfp were stimulated with the conditioned medium of interferon (IFN)-γ-activated HMC3 microglia, SGD inhibited the expression of inducible nitric oxide synthase (iNOS), NLRP1 and NLRP3, tumor necrosis factor (TNF)-α, interleukin (IL)-1β, and IL-6, attenuated caspase-1 activity and the production of ROS, and promoted neurite outgrowth. In streptozotocin-induced high-glucose APP/PS1/Tau triple transgenic (3×Tg-AD) mice, SGD also decreased the expression of NLRP1, NLRP3, Aβ, and Tau in the hippocampus and cortex, and improved working and spatial memory in the Y-maze and Morris water maze, thus demonstrating the potential of SGD in treating Alzheimer’s disease by alleviating neuroinflammation.

#### 4.1.3 Intestinal-brain axis protection

It was found that treatment with SGD reduced the release of pro-inflammatory cytokines and enhanced the expression of tight junction proteins (occludin and claudin1), thus preventing lipopolysaccharide (LPS) from entering the bloodstream. SGD could significantly decrease the ratio of Firmicutes to Bacteroidetes in PCOS rats, reduce the abundance of Proteobacteria, a LPS-producing pathogen, and enrich the abundances of Butyricicoccus, Coprococcus, Akkermansia Blautia, and *Bacteroides*. SGD inhibited the expression of key genes and proteins in the TLR4/NF-κB signaling pathway. Therefore, SGD may alleviate the inflammatory response in PCOS rats by remodeling the intestinal flora structure, protecting the intestinal barrier, and inhibiting the TLR4/NF-κB signaling pathway (Chang et al., 2021).

The above mechanism is relevant to the treatment of neurological diseases discussed in Section 3.4.

### 4.2 Anti-inflammatory effect

#### 4.2.1 Validation of classical inflammatory models

The principle of the carrageenan-induced inflammation model and the operation of the cotton ball implantation granuloma model are two classical animal experimental models commonly used to evaluate the anti-inflammatory activity of substances (especially drugs). The acetic acid-induced writhing test is one of the gold standard models for screening peripheral analgesic activity. It is mainly used to evaluate the peripheral analgesic activity of drugs (especially non-steroidal anti-inflammatory drugs and central analgesics). By combining the central analgesia model and anti-inflammatory experiments (such as carrageenan-induced paw swelling) for comprehensive interpretation, non-specific interference can be excluded and the mechanism of drug action can be clarified. Japanese scholars used the classical inflammatory models of carrageenan (acute inflammation) and the cotton ball method (chronic inflammation) and found that SGD has anti-inflammatory effects (Sugishita et al., 1984). The addition of Paeonia lactiflora reduced the anti-inflammatory effect of Glycyrrhiza uralensis; while in the acetic acid-induced writhing test, the addition of Glycyrrhiza uralensis enhanced the effect of Paeonia lactiflora.

#### 4.2.2 Molecular mechanisms

This includes regulation through signaling pathways and cytokine regulation. The SGD alleviates hyperandrogenemia in a letrozole-induced polycystic ovary rat model by inhibiting the syndrome caused by NF-κB activation (Shao et al., 2019). Research has shown the therapeutic potential of the SGD in alleviating anti-tuberculosis drug-induced liver injury through the Nrf-2/HO-1/nf-κB signaling pathway (Zhang et al., 2024).

The above mechanism is relevant to the treatment of immune system diseases discussed in Section 3.5.

### 4.3 Spasmolytic effect

Acetylcholine (ACh), as an important neurotransmitter, plays a central role in the regulation of muscle function. However, abnormal release or signal dysregulation of ACh can directly or indirectly lead to muscle pain. This pain mechanism involves the peripheral neuromuscular junction, central sensitization, and immune-neural interactions. SGD has the effect of relieving intestinal spasm and thus exerts antispasmodic and analgesic effects. Neurogenic contractions maintain regular intestinal peristalsis (such as migrating motor complexes); ACh-induced contractions are more involved in acute tonic contractions (such as inflammation or drug stimulation). Studies have found that SGD and its single botanical drugs (Paeonia lactiflora and Glycyrrhiza uralensis) inhibit the neurogenic contractions of the ileum induced by electrical stimulation and ganglionic stimulants such as DMPP and nicotine. SGD also has an inhibitory effect on ACh-induced contractions, thus playing an antispasmodic role (Maeda et al., 1983).

The above mechanisms are correlated with the analgesic effects and the treatment of digestive, skeletal and renal system diseases discussed in sections 3.1, 3.2, 3.6 and 3.7.

### 4.4 Other therapeutic potential

#### 4.4.1 Genes related to mental illness causing depression

Calculate and analyze the pathogenic genes of depression through bioinformatics methods. Through the research on the correlation between the targets of SGD, deduce and predict the key genes of SGD in the treatment of depression. By making LPS-induced depression model mice, drug treatment, behavioral tests, and detection of hippocampal tissue samples, it was found that SGD can regulate the levels of IL-10, TNF-α, BDNF, SMAD3, FGFR1, and FGFR2 to improve the depressive state, providing a theoretical basis for exploring the efficacy of SGD in the treatment of depression (Li L. et al., 2024).

#### 4.4.2 Oxygen deficiency protection

HIF1A (Hypoxia-Inducible Factor 1-Alpha) is a core transcription factor for cells to sense and respond to a hypoxic environment, playing a key role in pathophysiological processes such as tumor progression, inflammation, and metabolic reprogramming. HIF1A is a protective factor that inhibits compression-induced death of NPMSCs. Quercetin, a bioactive metabolite found in the traditional Chinese medicine formula SGD, improves the survival rate of NPMSCs and alleviates the progression of lumbar disc degeneration (LDD) by stabilizing HIF1A. Targeting the HIF1A pathway with natural metabolites such as quercetin may provide a promising strategy for the clinical treatment of LDD and potentially other degenerative disc diseases (Ren et al., 2024).

#### 4.4.3 MAPK signaling pathway

Three key active metabolites and eight core targets were screened out through network pharmacology analysis. The KEGG results showed that the PI3K/Akt and MAPK signaling pathways were the key signaling pathways for SGD in the treatment of gastric cancer. The experimental results indicated that SGD could inhibit the proliferation of AGS cells, induce apoptosis, arrest the cell cycle, and reduce the cell colony formation ability by regulating the PI3K/Akt and MAPK signaling pathways (Zhou et al., 2023).

#### 4.4.4 Ferroptosis

In ulcerative colitis, SGD decreased the disease activity index, levels of inflammatory factors, and histological damage in mice. Additionally, SGD downregulated the ferroptosis level of cells in the colon tissue, manifested as reduced iron overload, glutathione depletion, and a lower level of malondialdehyde production compared to the model group. Correspondingly, SGD had a similar inhibitory effect on the ferroptosis of Caco-2 cells treated with erastin (a ferroptosis inducer). The results of our *in vitro* reactive oxygen species assay and the mitochondrial structure observed by variable scanning electron microscopy also support these findings (Hu et al., 2023).

#### 4.4.5 Mitigation of autophagy

SGD alleviates APAP-induced mitochondrial damage, inflammation, and necrosis by promoting mitophagy. Inhibition of autophagy negates the hepatoprotective effect of SGD. SGD promotes autophagy/mitophagy and effectively mitigates APAP-induced hepatotoxicity, indicating the potential of SGD as a therapeutic agent for APAP-induced liver injury (Wu et al., 2024).

The above mechanisms are relevant to the treatment of digestive, skeletal and mental system diseases discussed in Sections 3.2, 3.6 and 3.10. The specific target/pathway are summarized in Table 1.

**TABLE 1:** Mechanism of action and multi - target regulation.

Target/Pathway	Function	References
cPLA2	Suppress the production of PG in the myometrium and relieve pain	Shibata et al. (1996)
NGF/TRPV1/COX-2	It exerts an analgesic effect in the rat model of arthritis pain	Sui and Tang (2016)
H-K-ATPase	Inhibit H-K -ATP to improve the pain of peptic ulcer	Satoh et al. (2001)
MAOB	Inhibiting MAOB alleviates *Helicobacter* pylori-induced chronic atrophic gastritis	Li et al. (2024a)
SCF/c-Kit	Upregulating the expression of c-Kit and SCF, activating the SCF/c-Kit pathway, thereby upregulating the expression of Bcl2, and inhibiting the expression of cleaved caspase-3, Bax, and tumor necrosis factor can treat SO dysfunction	Zhu et al. (2021)
CYP2E1	Improve the overexpression of CYP2E1, reduce oxidative stress, increase antioxidant enzymes (SOD, GSH), decrease peroxidation product MDA and inflammatory responses (IL-6, TNF-α), and alleviate liver tissue lesions	Li et al. (2022)
BAs metabolic pathway, bile acid/FXR	SGD can significantly change the mRNA expression of genes related to the BAs metabolic pathway, and improve dyslipidemia in PCOS rats by reshaping the gut microbiota structure and regulating the bile acid/FXR pathway	Duan et al. (2025)
PI3K/AKT	SGD can effectively upregulate the expression of PI3K and AKT proteins, which is related to the treatment of Alzheimer’s disease	Lv et al. (2022)
PI3K/AKT and NF-κB	The active metabolite of SGD, calycosin, may exert a protective effect against OA by inhibiting the PI3K/AKT and NF-κB pathways	Shi et al. (2022)
L-type calcium channel	SGD exerts a protective effect on AngⅡ-induced cardiomyocyte hypertrophy by inhibiting the L-Ca2+-mediated pathway	Tagashira et al. (2024)
MAPK signal pathway	Inhibit the proliferation of AGS cells, induce apoptosis, arrest the cell cycle, reduce the colony - forming ability of cells, and treat gastric cancer	Zhou et al. (2023)
TLR4/NF-κB	Remodeling the structure of intestinal flora, protecting the intestinal barrier, and improving the inflammatory response of rats	Chang et al. (2021)
Nrf-2/HO-1/nf-κb	Reduce anti - tuberculosis drug - induced liver injury	Zhang et al. (2024)
TRPV1 and TLR4-MyD88	Inhibit the overexpression of TRPV1 and TLR4-MyD88 signaling pathways to improve peripheral neuropathy	Chen et al. (2022)

## 5 Modern application expansion

### 5.1 Reduce the side effects of anti - tumor drugs

Paclitaxel-induced peripheral neuropathy (PIPN) has become one of the most common adverse reactions in the treatment of cancer patients and further promotes neuroinflammation in the nervous system. SGD has become a major adjuvant drug for chemotherapy to alleviate side effects. Especially in the case of PIPN, SGD has an analgesic effect on thermal hyperalgesia in the PIPN model. This protective effect is related to the inhibition of the overexpression of the TRPV1 and TLR4-MyD88 signaling pathways (Chen et al., 2022). Prophylactic topical application of paeoniflorin can prevent paclitaxel-induced mechanical hyperalgesia in mice through the adenosine A1 receptor (Andoh et al., 2017a). By increasing the proportion of phosphorylated Erk1/2 and phosphorylated Akt in pheochromocytoma PC12 cells, it promotes the axonal growth of NGF. This effect is applicable to the recovery of axonal involvement caused by paclitaxel and may promote the recovery of paclitaxel-induced neuropathy without affecting the anti-cancer effect of paclitaxel (Konaka et al., 2017).

SGD can effectively improve chemotherapy drug-induced neuralgia (Schröder et al., 2013), including improving paclitaxel-related very acute pain (Satoh, 2013). It can prevent joint pain and myalgia caused by the combination chemotherapy of paclitaxel and carboplatin (Fujiwara et al., 2000). Pretreatment with SGD can alter the pharmacokinetics of intravenously injected paclitaxel in rats (Wang et al., 2017). It has been reported that traditional Chinese medicine preparations such as SGD can inhibit drug-metabolizing enzymes and drug transporters, but the risk of drug interactions in patients receiving lenvatinib treatment is low. Patients should believe that they can receive traditional Chinese medicine preparations as supportive treatment for lenvatinib treatment without the risk of drug interactions that may affect efficacy (Fujita et al., 2025).

Peripheral neuropathy induced by oxaliplatin, especially cold sensory impairment, is the main dose-limiting side effect of this drug and is very difficult to control. TRPM8 is involved in oxaliplatin-induced cold sensory impairment. Repeated administration of SGD can inhibit the expression of TRPM8 mRNA in dorsal root ganglia induced by oxaliplatin. Prophylactic repeated administration of SGD can effectively prevent the aggravation of oxaliplatin-induced cold sensory impairment by inhibiting the mRNA expression of TRPM8 in dorsal root ganglia (Andoh et al., 2017b). It can reduce the neurotoxicity in colorectal cancer patients receiving oxaliplatin and FOLFOX (5-fluorouracil/leucovorin plus oxaliplatin) treatment (Okumi and Koyama, 2014). In colorectal cancer patients treated with the FOLFOX regimen, the application of traditional Chinese medicine metabolites may reduce the neurotoxicity caused by oxaliplatin without affecting the anti-tumor efficacy (Hosokawa et al., 2012). SGD has been standardized for the treatment of myalgia/arthritis caused by chemotherapeutic drugs in evidence-based medical research in Kampo medicine (Motoo, 2022). SGD also has the effect of inhibiting chemotherapy-induced diaphragmatic hiccups (Kamoshida et al., 2021).

SGD has certain clinical application value in the treatment of tetanus complicated with muscle spasm. By reducing the use of sedatives and analgesics, it can also prevent tetanus patients who require intensive care from entering the PICU (Oshima et al., 2023).

SGD can effectively improve olanzapine-induced hyperprolactinemia, has no significant effect on mental symptoms, and no obvious adverse reactions have been observed (Gu et al., 2016; Hori et al., 2013).

### 5.2 Dose alteration

The SGD can treat muscle spasms and lumbar spinal stenosis. The dosage indicates that the therapeutic effect of taking 2.5 g of detoxified traditional Chinese medicine as needed is equivalent to that of taking 7.5 g/d regularly (Takao et al., 2015). SGD is usually applied in a 1:1 ratio clinically, but studies have found that SGD in a 3:1 ratio can also effectively relieve spasms and pain (Wang et al., 2016). The use of clinical dosages warrants further research.

## 6 Safety and limitations

### 6.1 Safety

As a traditional Chinese medicine metabolite, SGD is widely used in clinical practice, and its safety has been extensively verified in thousands of years of clinical practice. Modern pharmacology indicates that, at recommended dosages, the use for less than 4 weeks is associated with relatively few adverse reactions. Common mild side effects include gastrointestinal discomfort and mild edema.

### 6.2 Limitations

Long - term and high - dose use (more than 8 weeks) may lead to pseudoaldosteronism, thus resulting in hypokalemia (Arai et al., 2023). There are also case reports indicating that drug - induced pneumonia occurred after taking SGD. After discontinuing the drug, the respiratory symptoms improved (Fujita et al., 2008).

## 7 Summary

The SGD is a paradigm for the “disease-syndrome combination” research. It is necessary to deeply analyze its multi-target mechanism in combination with systems pharmacology and metabolomics. The action targets of the SGD have been identified to be involved in the regulation of various biological activities, including calcium- and cytokine-mediated signal transduction, calcium ion concentration and homeostasis, the cellular behavior of muscle and neuronal cells, inflammatory responses, and responses to chemicals, cytokines, drugs, and oxidative stress. These targets are further enriched in various pain-related signaling pathways, including the PI3K-Akt, estrogen, ErbB, neurotrophin, neuroactive ligand-receptor interaction, HIF-1, serotonergic synapse, JAK-STAT, and cAMP pathways. Therefore, these data provide a systematic basis for understanding the molecular mechanism of the analgesic activity of botanical drugs.

The safety of traditional Chinese medicine is based on rational use, and its risks and benefits need to be scientifically weighed. For the clinical application of SGD, there is currently a lack of high-quality large-scale RCTs, which is worthy of further research. With the development of modern pharmacological and toxicological research, its complex metabolites and mechanisms can be more accurately analyzed, awaiting further research.

In the future, multiple directions can be focused on. For example, precision therapy: combining metabolomics and genomics to clarify the individualized medication regimens of SGD (SGD) for different populations. Dosage form innovation: developing nanosystems or sustained-release technologies to solve the problem of low oral bioavailability of glycoside metabolites (such as paeoniflorin). In-depth mechanism analysis: using organoids or single-cell sequencing technology to reveal the molecular targets of SGD in regulating specific cell pathways (such as stellate cell activation). International promotion: promoting SGD to be included in international management guidelines for related diseases through evidence-based medical research (such as multicenter RCT trials). Optimization of integrated traditional Chinese and Western medicine treatment plans: establishing a sound diagnosis and treatment plan integrating traditional Chinese and Western medicine for further international promotion.

Based on its traditional applications, SGD, combined with modern multi-omics technologies and novel drug delivery systems, is expected to become a model of “integrated traditional Chinese and Western medicine” treatment, providing safer and more effective solutions for complex diseases.
